# Design, Synthesis, and Biological Evaluation of Benzimidazole-Derived Biocompatible Copper(II) and Zinc(II) Complexes as Anticancer Chemotherapeutics

**DOI:** 10.3390/ijms19051492

**Published:** 2018-05-16

**Authors:** Mohamed F. AlAjmi, Afzal Hussain, Md. Tabish Rehman, Azmat Ali Khan, Perwez Alam Shaikh, Rais Ahmad Khan

**Affiliations:** 1 Department of Pharmacognosy, College of Pharmacy, King Saud University, P.O. Box 2457, Riyadh 11451, KSA; malajmii@ksu.edu.sa (M.F.A.); m.tabish.rehman@gmail.com (M.T.R.); aperwez@ksu.edu.sa (P.A.S.); 2 Department of Pharmaceutical Chemistry, College of Pharmacy, King Saud University, P.O. Box 2457, Riyadh 11451, KSA; azmatbiotech@gmail.com; 3 Department of Chemistry, College of Science, King Saud University, P.O. Box 2455, Riyadh 11451, KSA

**Keywords:** metal complexes, interaction with biomolecules, nuclease activity, cytotoxicity, toxicity

## Abstract

Herein, we have synthesized and characterized a new benzimidazole-derived “BnI” ligand and its copper(II) complex, [Cu(BnI)_2_], **1**, and zinc(II) complex, [Zn(BnI)_2_], **2**, using elemental analysis and various spectroscopic techniques. Interaction of complexes **1** and **2** with the biomolecules viz. HSA (human serum albumin) and DNA were studied using absorption titration, fluorescence techniques, and in silico molecular docking studies. The results exhibited the significant binding propensity of both complexes **1** and **2**, but complex **1** showed more avid binding to HSA and DNA. Also, the nuclease activity of **1** and **2** was analyzed for pBR322 DNA, and the results obtained confirmed the potential of the complexes to cleave DNA. Moreover, the mechanistic pathway was studied in the presence of various radical scavengers, which revealed that ROS (reactive oxygen species) are responsible for the nuclease activity in complex **1**, whereas in complex **2**, the possibility of hydrolytic cleavage also exists. Furthermore, the cytotoxicity of the ligand and complexes **1** and **2** were studied on a panel of five different human cancer cells, namely: HepG2, SK-MEL-1, HT018, HeLa, and MDA-MB 231, and compared with the standard drug, cisplatin. The results are quite promising against MDA-MB 231 (breast cancer cell line of **1**), with an IC_50_ value that is nearly the same as the standard drug. Apoptosis was induced by complex **1** on MDA-MB 231 cells predominantly as studied by flow cytometry (FACS). The adhesion and migration of cancer cells were also examined upon treatment of complexes **1** and **2**. Furthermore, the in vivo chronic toxicity profile of complexes **1** and **2** was also studied on all of the major organs of the mice, and found them to be less toxic. Thus, the results warrant further investigations of complex **1**.

## 1. Introduction

The serendipitous discovery of cisplatin [1] as an anticancer chemotherapeutic agent [2,3,4] swiftly marked significant breakthroughs in the field of metallodrugs as potential anticancer agents [5], and prompted several researchers and groups to explore the field of the metal-based chemotherapeutic [6]. Several potential metallodrugs were discovered since then and were used in clinics worldwide to treat cancers, starting from cisplatin, oxaliplatin, carboplatin, etc. However, the severe side effects and resistance by the cancer cells confine their clinical application widely [7]. To curb increasing resistance and the unbearable cost of treatment, it is a necessity to design potential alternatives.

Continual efforts have been made to design and develop non-classical metal-based chemotherapeutics. Among them, NAMI-A “(imidazole)RuCl_4_(DMSO)(imidazole)” and KP1019 “(indazole)RuCl_4_(DMSO)(indazole)” as ruthenium-centered potential anticancer drugs entered into the clinical trials, but still lead to side effects, and cost remains a hurdle. So, many scientists concentrated their attention on transition metals, particularly copper and zinc, to design novel metal-based anticancer agents. Both the metal ions are bio-essential elements that play a prominent role in the active site of many metalloproteins. Copper redox systems are involved in the catalytic process in the body [8,9]. Copper generates reactive oxygen species (ROS), endogenous DNA damage, and nucleobase affinity, and thus favors proliferative activity [10,11,12]. For growth and development, zinc ion is needed; it regulates the metabolism of the cells and is also a major regulatory ion in the metabolism of cells, which shrink the cardiotoxicity and hepatotoxicity that is associated with several anticancer drugs [13,14].

Furthermore, copper and zinc also possess affinity towards protein and DNA, although their propensity of binding mostly depends on the organic motifs. Thus, the role of the organic moiety/coordinated ligand is also pivotal. In this context, benzimidazole moieties are unique and interesting as they bear coordination potential for metal centers, planarity, and mimic the imidazole functions in proteins [15,16]. The benzimidazole core is also present inside the body as a component of Vitamin B_12_ [17]. The importance of this core can be understood by its presence in active drugs such as mebendazole (“Vermox” anthelmintic agents) [18]. The presence of biologically active pharmacophore, an imidazole ring, gives it versatility in possessing broad biological activities, from antihistaminic, antitubercular, anti-HIV, NSAIDs, antihypertensive to anticancer, etc. [19,20,21,22,23]. Thus, benzimidazole-derived moieties’ introduction to the drug design may enhance the biological activities.

Prompted by the impetus of the above findings, we have synthesized and characterized new copper(II) and zinc(II) complexes of the benzimidazole-derived organic motif. Further, we have studied their interaction with HSA (human serum albumin) and DNA using spectroscopic techniques and in silico molecular docking studies. Moreover, both complexes and the ligand were tested against five human cancer cell lines, and their chronic toxicity profile in vivo was studied.

## 2. Results and Discussion

### 2.1. Synthesis and Characterization

The tridentate biologically active pharmacophore, the benzimidazole-derived ligand, was synthesized by stirring the mixture of 2-aminobenzimdazole and 1-methyl-1*H*-imidazole-2-carbaldehyde in toluene as solvent and in the presence of glacial acetic acid as a catalyst at 80 °C for 12 h. The yellow precipitate was isolated via rotavapor and recrystallized in methanol as a yellow-crystalline material suitable for X-ray crystallography. This organic motif “BnI” was characterized by UV, Fourier transform infrared (FT-IR), NMR, and elemental analysis.

**Figure 1:**
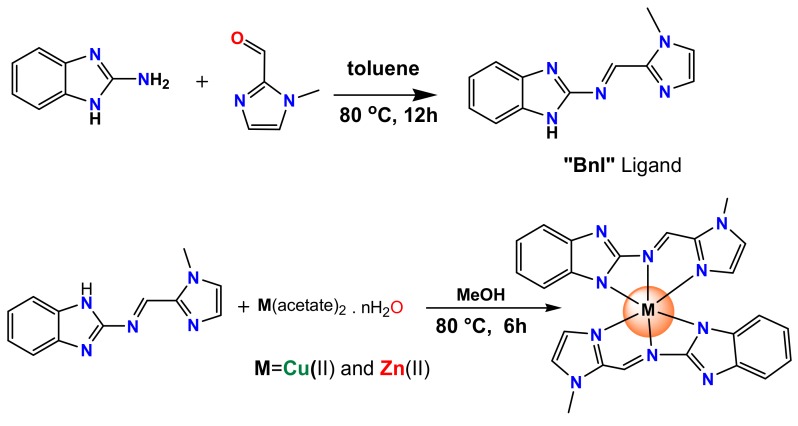
Schematic representation of the synthesis of the benzimidazole-derived ligand and its copper(II) complex [Cu(BnI)_2_], **1** and zinc(II) complex, [Zn(BnI)_2_], **2**.

The synthesis of complexes [Cu(BnI)_2_] **1** and [Zn(BnI)_2_] **2** was straightforward as the reaction was carried out between the ligand BnI and Cu(acetate)_2_·nH_2_O (**1**)/Zn(acetate)_2_·nH_2_O (**2**) in methanol solution in a molar ratio of 2:1, respectively (Figure 1).

Both the complexes **1** and **2** were isolated as green/yellow precipitate in reasonably good yield (73–86%). Complexes **1** and **2** were characterized by elemental microanalysis and various spectroscopic techniques. After several attempts, we failed to get crystals suitable for single X-ray crystallography. The electrospray ionization mass spectra (ESI-MS) of the complexes **1** and **2** revealed the presence of a molecular ion in significantly good agreement of the calculated and experimental *m*/*z* values.

The FT-IR spectrum of the ligand showed a characteristic strong peak at 1613 cm^−1^, which is associated with the C=N vibration of the Schiff base (Figure S1). Upon complexation with metal acetates, the FT-IR spectra of the complexes exhibited that both the signals of C=N vibrations got the significant shift to 1642 cm^−1^ for **1** (Figure S2) and 1644 cm^−1^ for **2** (Figure S3), thus confirming the coordination of the metal center with the ‘BnI’ ligand. The peak associated with M–N exhibited at 428 cm^−1^ and 438 cm^−1^ for complexes **1** and **2**, respectively (Figures S2 and S3).

The ^1^H NMR spectrum of the ligand exhibited a broad signals singlet for N–H of the benzimidazole ring at δ = 12.67 ppm (Figure S4). The characteristic signal for the aldimine proton HC=N was observed at 9.21 ppm, and the aromatic proton peak appeared in the range of 7.57–7.16 ppm for all six protons. Also, the singlet peak that was associated with the N–H_3_ protons appeared at 4.11 ppm. Upon complexation with Zn(OAc)_2_ (**2**) (Figure S5), the ^1^H NMR spectrum shows the absence of NH proton. Furthermore, the characteristic signal of aldimine protons gets shifted to 9.69 ppm and the aromatic protons also get shifted to a range of 7.24–6.84 ppm, which is suggestive of the coordination of the metal center with the nitrogens of the benzimidazole moiety and aldimine group. The protons of the imidazole ring appeared with a very little shift at 7.574 ppm from 7.571 ppm (0.003 ppm), thus confirming that the free movement of the imidazole ring may be because of the steric hindrance provided by the methyl group (N–CH_3_) over it. The peak associated with N–CH_3_ appeared at 3.92 ppm.

The ^13^C NMR spectra of the ligand and complex **2** further ascertain our proposed structure. The peak attributed to the ligand appeared in the range of 155.35 ppm for HC=N and 154.49–110.89 ppm for aromatic carbons, and at 35.42 ppm for the N–CH_3_ (Figure S6). Upon complexation with Zn(OAc), the organic moiety showed significant shifts in the signals. The characteristic signal associated with the aldimine carbon appears at 155.74 ppm (Figure S7). Other aromatic carbon peaks also get shifted and exhibited in the range of 143.29–120.44 ppm. The N–CH_3_ signal appeared at 64.90 ppm. Thus, the NMR of the complex **2** (Zn(II) complex) further confirmed the above-proposed structure.

The stability of the complexes **1** and **2** using absorption spectroscopy was studied in a dimethyl sulfoxide (DMSO) and phosphate buffer mixture that was incubated at room temperature and studied for 24 h. Both the complexes were quite stable; however, complex **2** showed signs of hydrolysis after 24 h.

### 2.2. HSA–Metal Complex Interaction by Spectroscopy

#### 2.2.1. Quenching Experiment

**Figure 2:**
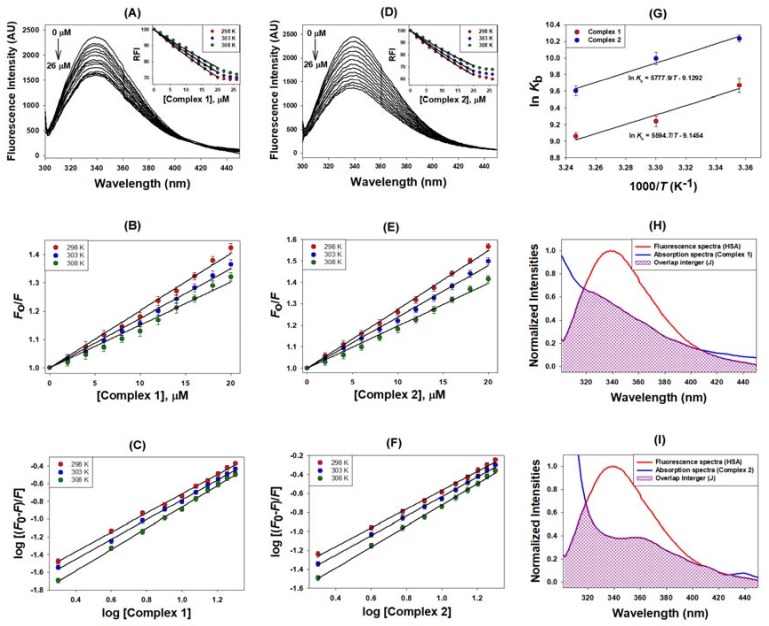
Molecular interaction between human serum albumin (HSA) and complexes **1** and **2**. (**A**,**D**) represent quenching in the fluorescence of HSA in the presence of complex **1** and **2**, respectively; (**B**,**E**) represent the Stern–Volmer plots of HSA-complex **1** and HSA-complex **2** respectively; (**C**,**F**) represent modified Stern–Volmer plots of HSA-complex **1** and HSA-complex **2** respectively; (**G**) represents the van’t Hoff plots for determining thermodynamic parameters; (**H**,**I**) represent FRET between HSA and complex **1** and **2**, respectively.

In this study, we followed quenching in human serum albumin (HSA) fluorescence emission in the presence of complexes **1** and **2** at different temperatures (298, 303, and 308 K). The emission intensity of HSA at 338 nm (*λ*_max_) was decreased significantly to 70% (Figure 2A and inset) and 63% (Figure 2D and inset) respectively for complexes **1** and **2** when the molar ratio of HSA:complex was 1:10. Stern–Volmer plots (Figure 2B,F) and the results presented in Table 1 shows that the *K*_SV_ values of the two complexes (**1** and **2**) were of the order of 10^4^ M^−1^, thereby indicating a very strong quenching phenomenon. The linear dependency of emission quenching as a function of the concentrations of complexes **1** and **2** suggested that only one type of quenching mechanism (either static or dynamic) was operational, and there was only one type of equivalent binding site on HSA for complex **1** and **2**. We also calculated the bimolecular quenching constant (*k_q_*) after taking the *τ_o_* of HSA as 5.71 × 10^−9^ s [24]. The *k_q_* values for **1** and **2** were of the order of 10^12^ M^−1^ s^−1^, which were at least 100 times more than the maximum collision constant of 10^10^ M^−1^ s^−1^ [25]. These results clearly indicate that the quenching of HSA emission intensity in the presence of complexes **1** and **2** was due to the formation of a complex rather than merely a collision event. Further, the type of quenching process that was involved was determined by evaluating the dependence of emission intensity quenching at different temperatures. The decreased values of *K*_SV_ and *k_q_* at elevated temperatures showed that a static quenching mechanism was involved in HSA and the systems of complexes **1** and **2** (Table 1). Furthermore, the slope of the modified Stern–Volmer plot indicated that both complexes **1** and **2** had only one type of binding site present on HSA (Figure 2C,F). Also, an intercept of the modified Stern–Volmer plot indicated that the binding constants of the two complexes **1** and **2** were of the magnitude of 10^4^ M^−1^, thus indicating a strong binding of complexes **1** and **2** with HSA (Table 1). Earlier studies also indicated that several ligands bind strongly to HSA with a binding constant ranging between 10^3^ M^−1^ to 10^5^ M^−1^ [26,27,28,29].

**Figure 3:**
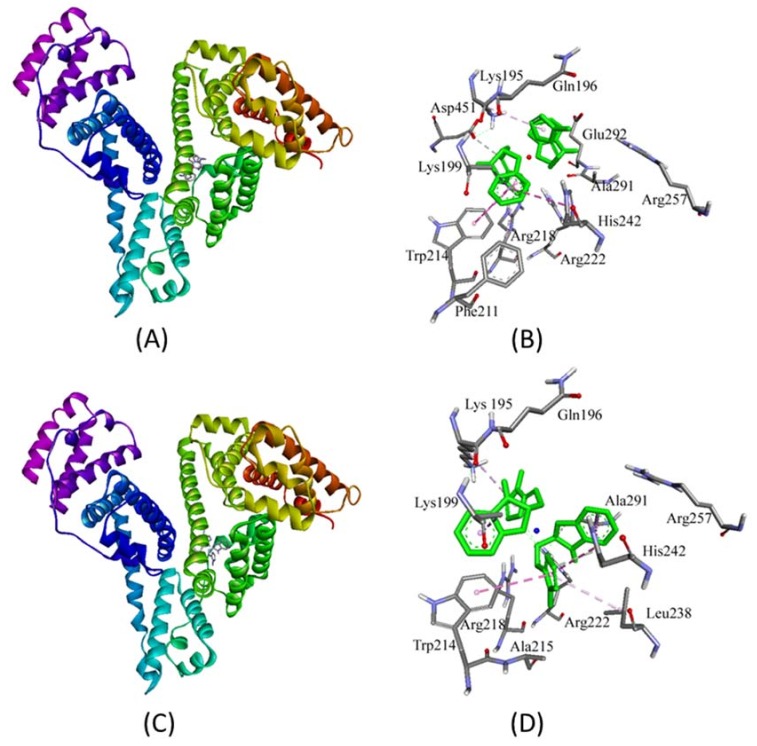
Molecular docking of complexes **1** and **2** with HSA. Panels (**A**,**C**) show the binding of complexes **1** and **2** near Sudlow’s site I in subdomain IIA respectively, and panels (**B**,**D**) represent the involvement of different amino acid residues of HSA in stabilizing complex with **1** and **2**, respectively. The amino acid residues are color coded according to element properties.

The thermodynamic parameters associated with the interaction between complexes **1** and **2** and HSA were estimated by assuming that the change in enthalpy (∆*H*) was not significant over the studied temperature range. Figure 3G shows the correlation between the binding constant (*K_a_*) and 1/*T*, the slope and intercept of which gives the values of −∆*H/R* and ∆*S/R*, respectively. We found that the values of ∆*H* and ∆*S* for the HSA-**1** interaction were −11.12 kcal/mol and −18.17 cal/mol/K, respectively. Similarly, for the HSA-**2** interaction, the ∆*H* and ∆*S* values were −11.48 kcal/mol and −18.14 cal/mol/K, respectively (Table 1). It is inferred from the above values that the enthalpy and entropy changes involved in the binding of complexes **1** and **2** to HSA were of a similar nature and magnitude. The negative values of ∆*H* and ∆*S* suggest that hydrogen bonding and van der Waal’s interactions were predominantly responsible for the formation of a stable HSA-**1** and HSA-**2** complex. Moreover, the interaction of HSA with complexes **1** and **2** was spontaneous, as suggested by the negative ∆*G* values (Table 1). At 298 K, the changes in Gibb’s free energy for HSA-**1** and HSA-**2** interactions were −5.71 kcal/mol and −6.08 kcal/mol, respectively.

#### 2.2.2. Förster Resonance Energy Transfer (FRET) between HSA and Metal Complexes

FRET occurs when the emission spectrum of the donor overlaps with the absorption spectrum of the acceptor [30]. Figure 2H,I depicts the overlap between the HSA fluorescence spectrum and the absorption spectrum of complexes **1** and **2**, respectively. We found that the efficiency of the energy transfer between HSA and the metal complexes was in the range of 30–37%. The value of *r* (distance between HSA and the metal complexes) for complexes **1** and **2** was 2.35 nm and 2.36 nm, respectively. Likewise, the values of *R_o_* (distance at which the efficiency of energy transfer becomes 50%) for complexes **1** and **2** with HSA interaction were 2.04 nm and 2.16 nm, respectively (Table 1). The deduced *r* values satisfied the condition of FRET that it should be within the 2–8 nm range. Moreover, the *r* values were within the range of 0.5*R_o_* < *r* < 1.5*R_o_*, thereby indicating that the quenching of HSA emissions was due to a complex formation between HSA and metal complexes (i.e., static quenching mechanism).

### 2.3. Molecular Docking Studies with HSA by Autodock

#### Prediction of Binding Sites of Metal Complexes on HSA

HSA is the most abundant protein in the plasma, and is responsible for the transportation of drugs. Three independent domains (I–III) are present in the structure of HSA. Domain I spans residues 1–195, domain II extends from residues 196–383, and domain III extends from residues 384–585; these are the basic HSA folds. Each domain has been divided into two subdomains, namely A and B [28]. The two principal binding sites of HSA are located in the hydrophobic cavities of subdomain IIA (Sudlow’s site I) and subdomain IIIA (Sudlow’s site II). A new binding site on HSA that is located at subdomain IB has been recently identified [31]. The binding site of metal complexes on HSA was predicted by an in silico approach using Autodock 4.2 software (The Scripps Research Institute, La Jolla, CA*,* USA). We found that both complexes **1** and **2** were bound to HSA at domain IIA near Sudlow’s site I mainly through hydrogen bonding and hydrophobic interactions (Table 2, Figure 3). In the HSA-**1** complex, a hydrogen bond (3.71 Å) between the H-atom of metal complex **1** and the O-atom of Asp451 was observed along with six hydrophobic interactions with Lys195, Lys199, Trp214, Arg218, and His242. Also, other key residues involved in stabilizing the HSA-**1** metal complex through van der Waal’s interactions were Gln196, Phe211, Arg218, Arg222, Arg257, Ala291, and Glu292. Similarly, complex **2** has been observed to interact with HSA by forming two hydrogen bonds with Ala291 and His242, and forming seven hydrophobic interactions with Lys195, Lys199, Trp214, Leu238, His242, and Ala291. Also, other residues involved in stabilizing the HSA-**1** complex through van der Waal’s interactions were Gln196, Ala215, Arg218, Arg222, and Arg257 (Table 2, Figure 3). The binding energy and Gibb’s free energy for complex **1** were 7.8 × 10^6^ M^−1^ and −9.4 kcal/mol, while for metal complex **2**, the binding affinity and Gibb’s free energy were 4.2 × 10^7^ M^−1^ and −10.4 kcal/mol, respectively. It is clear from the Table 2 and Figure 3 that complexes **1** and **2** were bound near Trp214, which explains the quenching of HSA fluorescence in the presence of these metal complexes. However, it should be noted that the values of ∆*G* obtained from in vitro binding experiments and computational prediction differ significantly. The ∆*G* value obtained in the in vitro experiment accurately measured the interaction between HSA and metal complexes in a solvent mimicking physiological conditions (i.e., it is global phenomenon). Conversely, the ∆*G* value acquired from the in silico docking experiment measured the interaction of metal complexes at the binding site of the protein only (i.e., it is a local phenomenon).

### 2.4. DNA Binding/Nuclease Activity

**Figure 4:**
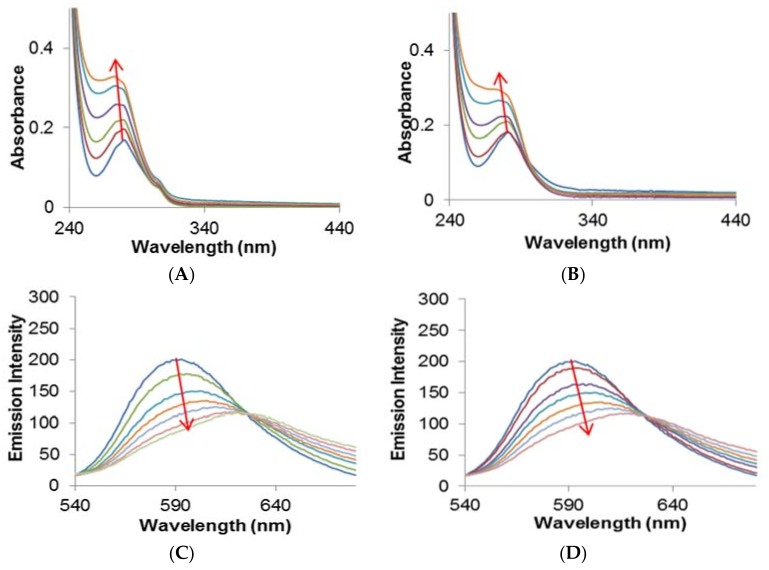
Absorption spectra of (**A**) complex **1**; and (**B**) complex **2** with CT-DNA. Fluorescence quenching spectra of (**C**) complex **1**; and (**D**) complex **2** with EthBr-DNA adduct. Both experiments were carried out in 5 mM Tris-HCl/50mM NaCl buffer, pH = 7.5, at room temperature. The spectra in different colors represent the effect of increasing concentrations (symbolized by an arrow) of complexes **1** and **2** on the studied spectroscopic properties.

DNA is thought to be one of the primary targets of the metallodrugs after cisplatin, which is an unanticipated finding for anticancer chemotherapeutics. Hence, we studied the binding affinity of complexes **1** and **2** with CT-DNA using spectroscopic studies such as absorption titration and an ethidium-bromide displacement assay. In the absorption studies, with the incremental addition of CT-DNA, the spectra exhibited hyperchromism with significant 6–9 nm blue-shifts, which is attributed to the binding of complexes **1** and **2** with CT-DNA via electrostatic mode (external groove) or partial intercalation (Figure 4A,B). Furthermore, the binding strengths were evaluated in the form of an intrinsic binding constant ‘*K_b_*’ using Wolfe–Shimer equation [32]. The *K_b_* values were found to be 2.67 × 10^4^ M^−1^ and 1.07 × 10^4^ M^−1^ for complexes **1** and **2**, respectively.

A further mode of interaction of complexes **1** and **2** with CT-DNA was studied via ethidium-bromide (EtBr) displacement assay, which involved competitive binding between a standard intercalator and complexes **1** and **2** against CT-DNA (Figure 4C,D). In this experiment, the pre-treated CT-DNA with EtBr was used in the standard ratio (EtBr is weakly emissive, whereas the DNA+EtBr adduct exhibits high emission intensity). The DNA-EtBr adduct was treated with an increasing concentration of complexes **1** and **2**; the results give rise to a significant reduction in the fluorescence intensity but don’t diminish the emission intensity 100%. These results ascertain that complexes **1** and **2** are not typical intercalators, but the possibilities of partial intercalation cannot be ruled out. The extent of quenching was evaluated by using the Stern–Volmer equation, and the *K*_SV_ values were calculated and were found to be 2.03 M^−1^ and 1.21 M^−1^ for complexes **1** and **2**, respectively.

**Figure 5:**
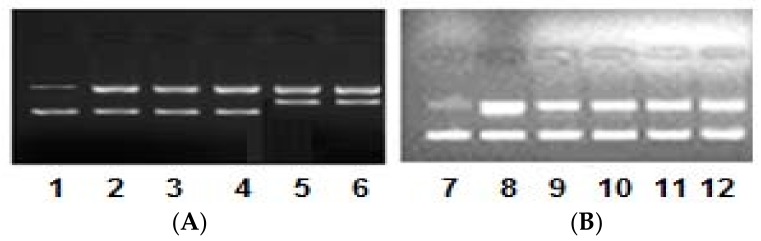
Electrophoretic pattern of pBR322 DNA (100 ng) by (**A**) complex **1** and (**B**) complex **2** (0.5–2.5 μM) in 50 mM Tris-HCl/NaCl buffer pH, 7.4 after 45 min of incubation at various concentrations. Lane 1: DNA alone (control); Lane 2: DNA + 0.5 μM **1**; Lane 3: DNA + 1.0 μM **1**; Lane 4: DNA + 1.5 μM **1**; Lane 5: DNA + 2.0 μM **1**; Lane 6: DNA + 2.5 μM **1**; Lane 7: DNA alone (control); Lane 8: DNA + 0.5 μM **2**; Lane 9: DNA + 1.0 μM **2**; Lane 10: DNA + 1.5 μM **2**; Lane 11: DNA + 2.0 μM **2**; Lane 12: DNA + 2.5 μM **2**.

We have further evaluated the nuclease activity of complexes **1** and **2** against pBR322 DNA. The concentration-dependent cleavage pattern was obtained with an increasing concentration of complexes **1** and **2** (0.5–2.5 μM) with pBR322 DNA (100 μM) (Figure 5).

**Figure 6:**
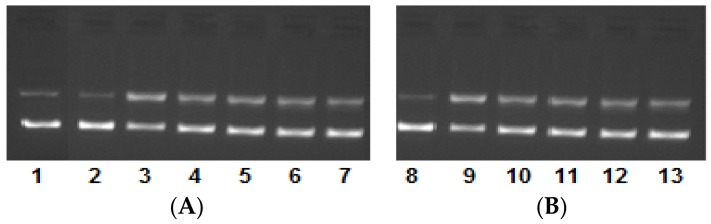
Mechanistic electrophoretic pattern of pBR322 DNA (100 ng) by (**A**) complex **1** and (**B**) complex **2** (0.5–2.5 μM) in 50 mM of Tris-HCl/NaCl buffer pH 7.4 after 45 min of incubation at various concentrations. Lane 1: DNA alone (control); Lane 2: DNA + DAPI + **1**; Lane 3: DNA + MG + **1**; Lane 4: DNA + DMSO + **1**; Lane 5: DNA + EtOH + **1**; Lane 6: DNA + NaN_3_ + **1**; Lane 7: DNA + SOD+ **1**; Lane 8: DNA + DAPI + **2**; Lane 9: DNA + MG + **2**; Lane 10: DNA + DMSO + **2**; Lane 11: DNA + EtOH + **2**; Lane 12: DNA + NaN_3_ + **2**; Lane 13: DNA + SOD + **2**.

The electrophoretic pattern that was observed exhibited significantly good cleavage with complex **1**, and was exhibited by the appearance of form III (linear form from the supercoiled circular ‘native form’), which ascertained the double-stranded incision of the DNA. However, complex **2** displayed moderate cleavage activity, giving rise to form II (nicked circular form), which resulted due to the single-stranded incision of the DNA. However, the appearance of the broad band swelling of bands in the presence of complex **2** ascertains the hydrolysis of the DNA. Furthermore, the mechanistic pathway of the DNA cleavage by complexes **1** and **2** was also examined, and the results are presented in Figure 6. In this experiment, the DNA and complex reaction mixture were treated with radical scavengers (hydroxyl radical scavengers viz., DMSO, EtOH), a singlet oxygen radical scavenger (viz., NaN_3_) and a superoxide radical scavenger (viz., SOD). The electrophoretic pattern observed confirms that the generation of hydroxyl radical, singlet oxygen radical, and superoxide radicals are the responsible species for the cleavage of the DNA. Thus, both complexes **1** and **2** cleave the DNA by the freely diffusible radical mechanism. At higher concentrations, that complex **2** exhibits hydrolytic cleavage is quite possible. To get into the insight of the DNA groove-binding propensity of complexes **1** and **2**, the DNA cleavage was also carried out in the presence of 4',6-diamidino-2-phenylindole (DAPI, minor groove binder) and methyl green (MG, major groove binder) standards. The mixture of DNA and known groove binders were treated with the complexes and the results exhibited that complexes **1** and **2** possess the minor groove-binding tendency (Figure 6).

### 2.5. Molecular Docking Studies with DNA by Hex

#### Prediction of Binding Sites of Metal Complexes on DNA

**Figure 7:**
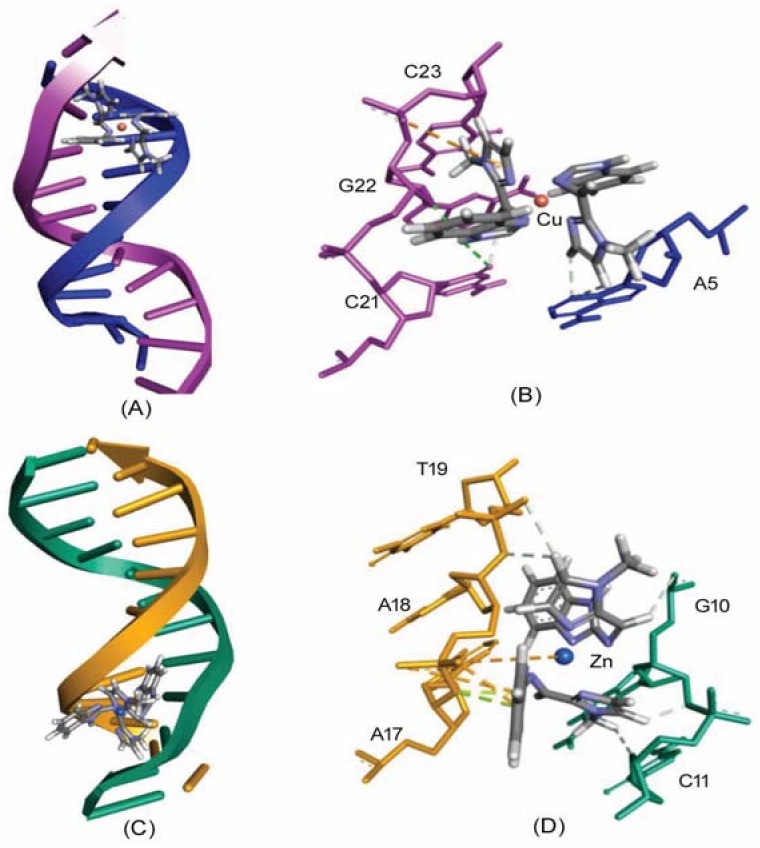
Molecular docking of complexes **1** and **2** with DNA. Panels (**A**,**C**) represent the binding of complexes **1** and **2** at the major groove of DNA, respectively; and panels (**B**,**D**) depict the residues of DNA making contacts with complexes **1** and **2**, respectively. Different strands and nucleotides of DNA are represented by separate color (Magenta and Blue in (**A**,**B**), and Green and Yellow in (**C**,**D**)). Metal complexes **1** and **2** are represented according to element composition.

In the present study, we have used Hex 8.0 software (Dave Ritchie, Capsid research team at the LORIA/Inria, Nancy, France) to probe the binding site of complexes **1** and **2** on the dodecameric DNA molecule, and the molecular interactions involved in stabilizing the complex (Table 3, Figure 7).

Findings of the docking results further support our in vitro results that the most preferred binding site of complexes **1** and **2** on DNA was located at the minor groove of DNA, with an overall binding score of −238.14 and −248.34, respectively. It was bound in a GC-rich region in such a fashion that allowed the planar part of the molecule to form favorable interactions with the nitrogenous bases of the DNA molecule (Table 3).

The DNA-complex **1** adduct was stabilized by five hydrogen bonds (dA5:N3 of chain A and d21:O2 of chain B formed two hydrogen bonds each with complex **1,** while dG22:O4′ formed one hydrogen bond with complex **1**). Moreover, the DNA-complex **1** adduct was stabilized by one electrostatic interaction with d23:OP1 of chain B. Similarly, complex **2** formed three electrostatic interactions and five hydrogen bonds along with two π-lone pair interactions with DNA molecules (Table 3, Figure 7). The metal ligand Zn was electrostatically involved with dA18:OP1 of chain B. Also, dA18:OP1 of chain B formed two electrostatic interactions, while dA17:O3′ of chain B formed two π-lone pair interactions. Moreover, the overall DNA-complex **2** adduct was further stabilized by five hydrogen bonds with d10:O3′, dG10:O4′, and d11:O4′ of chain A, and dA18:O3′ and dT19:OP1 of chain B.

### 2.6. In Vitro Cytotoxicity Assays

#### 2.6.1. Analysis of Growth Inhibition Using 5-Diphenyltetrazolium Bromide (MTT) Assay

In view of the above findings, the two novel complexes **1** and **2** were investigated for their anticancer efficacy in vitro. The cytotoxic effect of the synthesized complexes was examined on a panel of cancer cells. The cytotoxic efficacy was expressed in terms of IC_50_ values, as shown in Table 4. The newly synthesized complex **1** inhibited the cell growth of all the five tested cell lines in a dose-dependent manner. MDA-MB 231 was observed to be the most sensitive to complex **1**, followed by HeLa > HepG2 > HT108 > SK-MEL-1. On the other hand, the novel complex **2** inhibited the cell growth of four out of five tested cell lines in a dose-dependent manner. Complex **2** was most effective on SK-MEL-1 followed by HepG2 > HeLa > HT108. However, complex **2** showed the least effect on MDA-MB 231 (Table 4). In comparison with complex **2**, complex **1** showed potential in vitro anticancer activity against all of the tested cancer cell lines. However, no activity against particular cancer cell lines was observed with free ligands. Nevertheless, the anticancer potential of complex **1** was due to the presence of its copper moiety, which efficiently inhibited the growth of cancer cells. Considering this, we continued our anticancer study further with complex **1** only. Complex **1** showed significant activity when compared with standard drugs and some of the previously reported copper(II) complexes. For example, the copper(II) complex of 4′-methoxy-5,7-dihydroxy-isoflavone exhibited cytotoxic effects against cancer cell lines viz., A549, HeLa, HepG2, SW620, and MDA-MB-435), with IC_50_ values in the 10−50 μM range [33]. Also, the copper complexes [Cu(L)Cl(H_2_O)]Cl·3H_2_O of 2-methyl-1*H*-benzimidazole-5-carbohydrazide (L) displayed cytotoxicity against A549 cancer cells (IC_50_ = 20 μM) [34].

#### 2.6.2. Effect on Cancer Cell Adhesion/Migration

**Figure 8:**
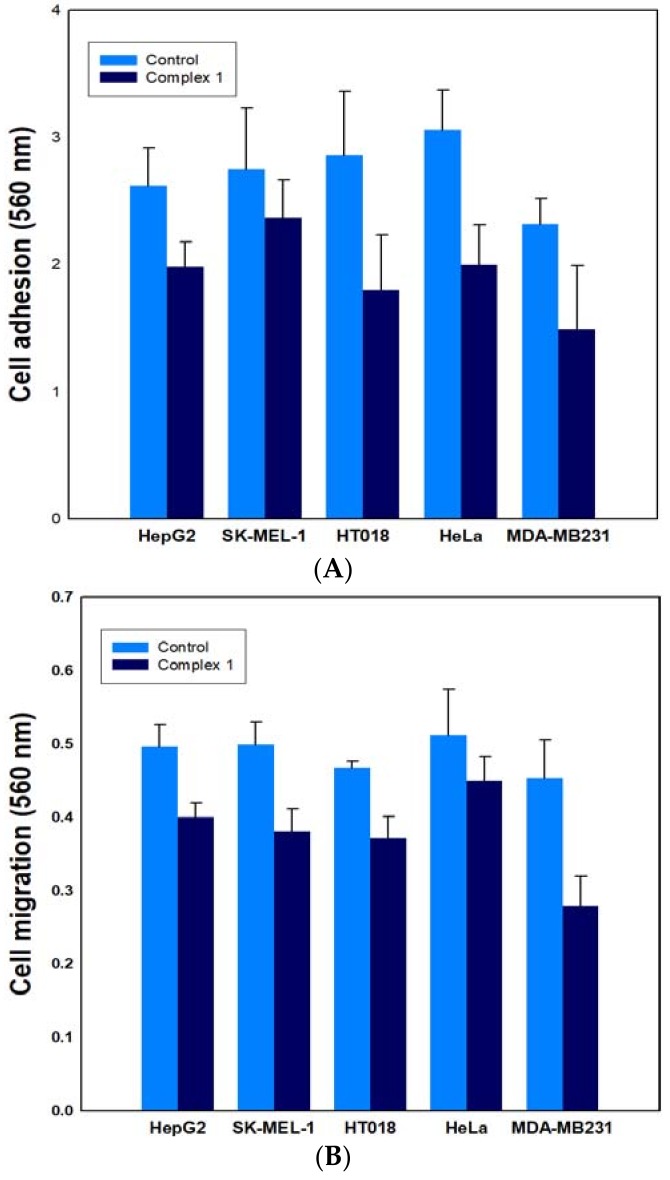
Effect of complex **1** on (**A**) cell adhesion and (**B**) cell migration against five cancer cell lines. Assays for adhesion and migration were performed with a cytoselect 24-well plate, and the absorbance of extracted samples was read at 560 nm.

Cancer metastasis is the spread of cancer cells to tissues and organs far from where a tumor originated. Cancer cells must be able to stick together, move, and migrate in order to spread. The properties of cell adhesion and cell migration are fundamental in regulating cell movement and cancer metastasis, and are necessary for the cells to move into the bloodstream. To further characterize the anticancer activity of the potent complex **1**, its inhibitory effect was investigated on the adhesion and migration of cancer cells at an IC_50_ concentration for the respective cancer cell lines. Complex **1** was tested at concentrations of 14 μM, 17.8 μM, 15 μM, 13 μM, and 3.5 μM against HepG2, SK-MEL-1, HT 018, HeLa, and MDA-MB 231, respectively. We found that complex **1** inhibited cell adhesion by about 16.4% (14 μM), 21.3% (17.8 μM), 16.5% (15 μM), 12.2% (13 μM), and 35.2% (3.5 μM) of HepG2, SK-MEL-1, HT 018, HeLa, and MDA-MB 231, respectively (Figure 8A). As far as migration is concerned, complex **1** inhibited the migration phenomenon of the tested cancer cells. However, the rate of inhibition varied with the respective cell lines, respectively (Figure 8B). Therefore, we conclude that complex **1** was able to affect the metastatic potential of cancer cells by inhibiting their adhesion as well as migration properties.

#### 2.6.3. Annexin V Apoptosis Detection Assay

**Figure 9:**
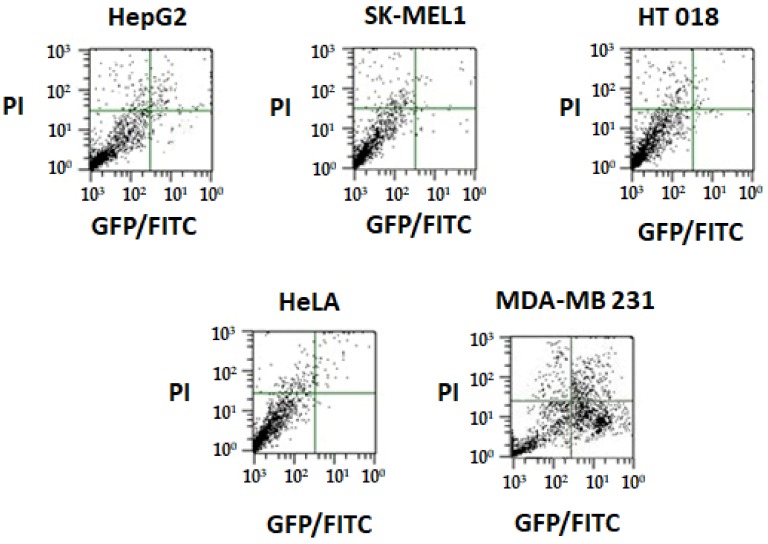
The apoptotic effect of complex **1** using annexin-V staining of tested cancer cell lines. Dots represent cells as follows: lower left quadrant, normal cells (FITC^−^/PI^−^); lower right quadrant, early apoptotic cells (FITC^+^/PI^−^); upper left quadrant, necrotic cells (FITC^−^/PI^+^); upper right quadrant, late apoptotic cells (FITC^+^/PI^+^).

Apoptosis and necrosis are two primary mechanisms of cell death. Cells that are damaged by external injury undergo necrosis, while cells that are induced to commit programmed suicide because of internal or external stimuli undergo apoptosis. An increasing number of chemopreventive agents have been shown to stimulate apoptosis in pre-malignant and malignant cells in vitro or in vivo [35]. A gross majority of classical apoptotic attributes can be quantitatively examined by flow cytometry (FACS). The apoptotic effect of complex **1** was evaluated using annexin-V staining. All of the tested cancer cells were harvested after treatment by complex **1** for 48 h and incubated with annexin V-FITC and PI. First, 10,000 cells were analyzed per determination. Dot plots show annexin V–FITC binding on the *X*-axis and propidium iodide (PI) staining on the *Y*-axis (Figure 9).

Data of all of the cell lines after treatment with complex **1** showed a decrease in viable cancer cells. Distinct 3.9% (HepG2), 1.9% (SK-MEL-1), 1.0% (HT018), 0.2% (HeLa), and 32.6% (MDA-MB 231) increases in apoptotic cell population was recorded. After 48 h of exposure time, due to late apoptosis, the cell population increased to 7.2%, 2.7%, 1.2%, 1.5%, and 26.1% for HepG2, SK-MEL-1, HT018, HeLa, and MDA-MB 231, respectively. We found that complex **1** was the most effective on MDA-MB-231 cells. The cytotoxic effect of the complex was not correlated to the increase of the necrotic cell population.

### 2.7. In Vivo Chronic Toxicity Studies of Complex **1** vs. **2**

**Figure 10:**
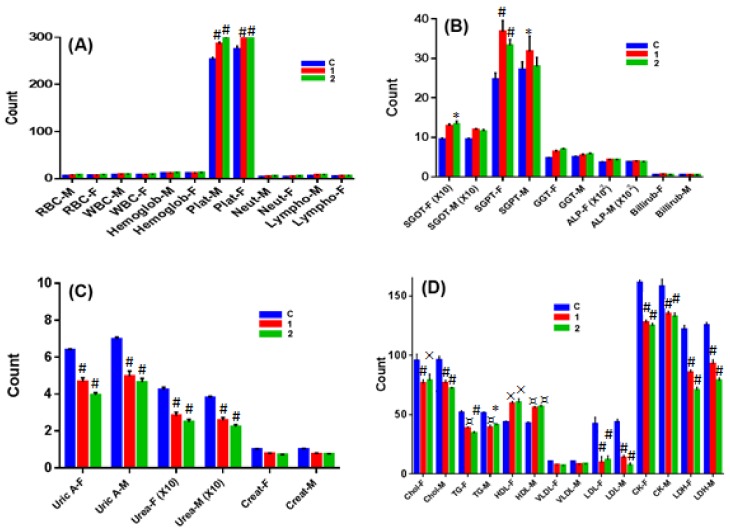
Chronic toxicity profile of complexes **1** and **2**. Effect of complexes **1** and **2** on (**A**) blood components; (**B**) biochemical parameters of liver function; (**C**) biochemical parameters of kidney function; and (**D**) biochemical parameters of lipids and heart function. M and F denote male and female mice, respectively. Statistical analysis was performed by two-way ANOVA, as well as the Dunnett test as the post-hoc test, compared with control (**C**), *n* = 4. (* *p* < 0.05; ¤ *p* < 0.01; × *p* < 0.005; # *p* < 0.001).

To evaluate the safety of complexes **1** and **2**, we studied their chronic toxicity in male as well as female mice. The hematology studies revealed that both complexes **1** and **2** increased the count of red blood cells (RBC) and white blood cells (WBC) in male mice, as well as hemoglobin, platelets, neutrophils, and lymphocytes, with neutrophils and lymphocytes being the most significantly affected cells (Figure 10A). Complex **2** was more potent in increasing the count of these cells than complex **1**. This might be due to the stimulation of bone marrow or increased protection of it from the deleterious effects, leading to the production of cells. In female mice, complex **1** showed weak activity against the above-mentioned cells except for neutrophils and lymphocytes, where it possessed significantly increased initial counts. Although both complexes significantly increased the clotting time in both male and female mice, complex 2 seemed to be more potent. Interestingly, both complexes increased fertility in mice. Both had reduced dead fetus percentages at the end of term, with complex **2** having a far lower dead fetus percentage.

In liver function tests (Figure 10B), both complexes did not alter liver functions in male animals except SGOT (Serum Glutamic Oxaloacetic Transaminase), which was significantly elevated by both complexes. Both complexes significantly lowered blood glucose levels, with complex **2** possessing stronger blood sugar lowering activity. However, both complexes significantly elevated SGOT, SGPT (Serum Glutamic Pyruvic Transaminase), GGT (Gamma-Glutamyl Transferase), ALP (Alkaline Phosphatase), and bilirubin in female animals. Moreover, both complexes significantly decreased blood glucose levels.

In renal function tests (Figure 10C), both complexes significantly increased sodium and potassium levels, but decreased calcium, urea, uric acid, and blood creatinine in male mice to significant (and potent) levels. Both complexes possessed the same activity in female mice, but decreased potassium blood levels.

The lipid profile and protein tests (Figure 10D) confirmed that both the complexes decreased significant levels of blood triglycerides, cholesterol, LDL (Low Density Lipoproteins), and VLDL (Very Low Density Lipoproteins), but significantly increased the blood levels of HDL and total proteins in both male and female mice. In cardiac function tests (Figure 10D), both complexes significantly lowered the blood levels of LDH and creatine kinase in both male and female mice. Complex **2** showed stronger activity on both enzymes.

## 3. Experimental Section

### 3.1. Material

First, 2-aminobenzimidazole, 1-methyl-1*H*-imidazole-2-carbaldehyde, Cu(CH_3_COO)_2_·H_2_O, Zn(CH_3_COO)_2_·2H_2_O, pBR322 DNA, sodium azide, superoxide dismutase, H_2_O_2_, DAPI, methyl green (MG), the sodium salt of CT-DNA, and HSA essentially fatty acid-free (≥98%) were purchased from Sigma-Aldrich (St. Louis, MO, USA). *Tris*(hydroxymethyl)aminomethane hydrochloride (Tris-HCl) was of analytical grade and also obtained from Sigma-Aldrich (St. Louis, MO, USA). Rosewell Park Memorial Institute (RPMI)-1640 medium, Dulbecco’s minimum essential medium (DMEM), fetal calf serum (FCS), dimethyl sulphoxide (DMSO), trypsin-EDTA solution, dithiothreitol (DTT); 3-4,5-dimethylthiazol-2-yl-2, 5-diphenyltetrazolium bromide (MTT), and cisplatin were procured from Sigma-Aldrich (St. Louis, MO, USA). All of the other chemicals used were also of the highest purity. Cell adhesion and cell migration kits were obtained from Cell Biolabs, Inc (San Diego, CA, USA). A Vybrant Apoptosis Assay Kit was procured from Molecular Probe (Eugene, OR, USA). Solvents were used as received.

### 3.2. Synthesis Procedure of Schiff Base

A mixture of 2-aminobenzimidazole (5.0 mmol) and 1-aminoimizole-2-carboxyaldehyde (5.0 mmol) in 25 mL of toluene by adding a few drops of acetic acid was stirred at 80 °C for 12 h. The yellow coloration of the mixture deepened, and after 12 h of stirring, the solvent was removed by rotavapor. A yellow-colored compound was isolated. To recrystallize the compound, it was dissolved in MeOH, and the resulting solution was kept for slow evaporation. Yellow single crystals were isolated after two days. Yield: (68%). M.p. 265 °C. Anal. Calc. for 1_2_H_11_N_5_: C, 63.99; H, 4.92; N, 31.09. Found: C, 63.86; H, 4.89; N, 31.05%. FT-IR (KBr pellet, cm^−1^): 3384, 3050, 2979, 1612, 1565, 1463, 1427, 1380, 1279, 1159, 740. ^1^H NMR (CDCl_3_, δ, 293 K): 12.67 (s, 1H, 1NH), 9.21 (s, 1H, HC=N), 7.51–7.16 (ArH, 6H), 4.11 (s, 3H, N–CH_3_), ESI-MS: 226.1.

### 3.3. Copper Complex: [Cu(BnI)_2_], *(**1**)*

Yield: (86%). M.p.198 °C. Anal. Calc. for C_24_H_20_N_10_Cu: C, 56.30; H, 3.94; N, 27.36. Found: C, 56.46; H, 4.03; N, 27.29%. ESI MS (+ve) DMSO, m/z: 515.0 for C_24_H_20_N_10_Cu + 3H^+^. FT IR (KBr pellet, cm^−1^): 3408, 3062, 1642, 1597, 1469, 1390, 1272, 742, 672, 662, 507, 438. Λ_M_ (1 × 10^−3^ M, DMSO): 15.90 Ω^−1^ cm^2^ mol^−1^ (non-electrolyte in nature). UV-vis (1 × 10^−3^ M, DMSO, λ_nm_): 656 nm. μ_eff_ (B.M.) = 1.89. EPR (solid state, g_av_, 298 K) = 2.09.

### 3.4. Zinc Complex, [Zn(BnI)_2_], *(**2**)*

Yield: (73%). M.p. 174 °C. Anal. Calc. for C_24_H_20_N_10_Zn: C, 56.09; H, 3.92; N, 27.26. Found: C, 55.86; H, 3.89; N, 27.21%. ESI-MS (+ve) DMSO, m/z: 516.2 for C_24_H_20_N_10_Zn + 2H^+^. FT IR (KBr pellet, cm^−1^): 3408, 3055, 1644, 1582, 1466, 1392, 1271, 741, 668, 647, 512, 428. ^1^H NMR (DMSO, δ, 293 K): 9.68 (s, 2H, HC=N), 7.58-6.84 (ArH, 12H), 3.92 (s, 6H, N–CH_3_).

### 3.5. Fluorescence Quenching Measurements

Samples of HSA were prepared in 20 mM of sodium phosphate buffer (pH 7.4), while the 1-mM stock solution of complexes **1** and **2** in 10% DMSO was prepared and further diluted in 20 mM of sodium phosphate buffer to reach the desired concentration. In all of the samples, the final concentration of DMSO was below 1%. The concentration of HSA was determined from Beer–Lambert’s law using the molar extinction coefficient of 36,500 M^−1^ cm^−1^ at 280 nm [36].

Quenching in the fluorescence emission intensity of HSA was measured on a JASCO spectrofluorometer (FP-8300) fitted with a thermostatically controlled cell holder attached to a water bath. To the HSA sample, (2 μM, 3 mL) 2 μM of complexes **1** and **2** was successively added in such a manner that the total volume of metal complexes added was not more than 30 μL. Fluorescence quenching was measured by exciting Trp214 of HSA at 295 nm, and the fluorescence emission spectrum was collected between 300–450 nm [27]. Excitation and emission slits were kept at 5 nm. All of the fluorescence emission intensities were corrected for the inner filter effect using the following equation:

(1) $$F_{Corr}=F_{Obs}\times e^{(A_{ex}+A_{em})/2}$$

The quenching parameters were deduced using the following Stern–Volmer (Equation (2)) and modified Stern–Volmer (Equation (3)) equations:

(2) $$\frac{F_{o}}{F}=1+K_{SV}[Q]=1+k_{q}\tau_{o}[Q]$$

where *Fo* and *F* are the fluorescence emission intensities of HSA before and after metal complex binding; *K*_SV_ is the Stern–Volmer constant; [*Q*] is the molar concentration of the quencher, i.e., metal complex; *k_q_* is the bimolecular quenching rate constant, and *τ_o_* is the lifetime of HSA fluorescence in the absence of any quencher.

(3) $$\log\frac{\left(F_{o}-F\right)}{F}=\log K_{a}+n\log[Q]$$

where *Fo* and *F* are the fluorescence intensities of HSA before and after metal complex binding; *K_a_* is the binding constant; *n* is the number of binding sites, and [*Q*] is the molar concentration of the quencher.

The thermodynamic parameters for the interactions between HSA and complexes **1** and **2** were determined by measuring emission quenching at three different temperatures (298, 303, and 308 K). The following van’t Hoff and thermodynamic equations (equations (4) and (5)) were used to deduce the change in enthalpy (∆*H*), entropy (∆*S*), and Gibb’s free energy (∆*G*).

(4) $$\ln K_{a}=\frac{\Delta S}{R}-\frac{\Delta H}{RT}$$

(5) ΔG=ΔH−TΔS

### 3.6. FRET Measurements

The FRET between HSA and complexes **1** and **2** were observed by measuring the absorption spectra of complexes **1** and **2**, and the emission spectra of HSA in the 300–450 nm range. All of the intensities were normalized as described earlier [30]. The distance (*r)* between Trp214 of HSA and bound complexes **1** and **2** was measured using the following equations:

(6) $$E=\frac{R_{o}^{6}}{R_{o}^{6}+r^{6}}=1-\frac{F}{F_{o}}$$

(7) $$R_{o}^{6}=8.79\times 10^{-25}K^{2}n^{-4}\phi J$$

where *E* is the efficiency of the energy transfer; *R_o_* is the distance at which the efficiency of energy transfer becomes 50%; *r* is the distance between HSA (donor) and the metal complex (acceptor); *F* and *F_o_* are the emission intensities of HSA in the presence and absence of the metal complex (quencher); *K*^2^ is the geometry of the dipoles (*K*^2^ = 2/3 for HSA); *n* is the refractive index of the medium (here, it is 1.33); *φ* is the emission quantum yield of HSA in the absence of quencher (*φ* = 0.118), and *J* is the overlap integral of the HSA emission spectrum and the absorption spectrum of the metal complex.

The overlap integral (*J*) is determined from the following equation:

(8) $$J=\frac{\int_{0}^{\infty }F_{\lambda }\varepsilon_{\lambda }\lambda^{4}d\lambda }{\int_{0}^{\infty }F_{\lambda }d\lambda }$$

where *F_λ_* is the fluorescence intensity of HSA at the wavelength *λ*; and *ε_λ_* is the molar extinction coefficient of the metal complex at the wavelength *λ*.

### 3.7. DNA Binding and Nuclease Activity

The standard protocol [32] was adopted for both the experiments, with the slight modifications that we reported earlier [36,37,38,39,40].

### 3.8. Molecular Docking Studies

#### 3.8.1. Preparation of Ligands and Receptors

The three-dimensional structures of HSA (PDB Id:1AO6) and B-DNA dodecamer d(CGCGAATTCGCG)_2_ (PDB Id:1BNA) that were used for docking were retrieved from the RCSB PDB database bank [41]. Structures of the ligands (complexes **1** and **2**) were drawn in CHEMSKETCH (Available online: http://www.acdlabs.com) and converted to a PDB file using OPENBABEL (Available online: http://www.vcclab.org/lab/babel). The energies of complexes **1** and **2** were minimized using a MMFF94 forcefield. The water molecules and any heteroatoms were deleted from the receptor files (i.e., HSA and DNA) before setting up the docking program.

#### 3.8.2. Molecular Docking of Complexes **1** and **2** with DNA

HEX 8.0.0 software was used to study the binding of complexes **1** and **2** with a DNA molecule with default settings. The binding site of the ligand on the receptor was searched on the basis of shape as well as electrostatics, and the post-processing was done by optimized potentials for liquid simulations (OPLS) minimization. The GRID dimension was set at 0.6, and 10,000 solutions were computed. The results were analyzed in Discovery Studio 4.0 (Accelrys Software Inc., San Diago, USA, 2013) [42].

#### 3.8.3. Molecular Docking of Complexes **1** and **2** with HSA

Autodock4.2 was used for the docking of complexes 1 and 2 with HSA as described previously [27]. Briefly, Gasteiger partial charges were added to the atoms of complexes 1 and 2. Non-polar hydrogen atoms were merged, and rotatable bonds were defined. Essential hydrogen atoms, Kollman united-atom charges, and solvation parameters were added to HSA with the aid of the AutoDockTool. Affinity grid maps of 120 × 120 × 120 Å grid points and 0.7 Å spacing were generated using the AutoGrid program. Docking simulations were performed using the Lamarckian genetic algorithm (LGA) and the Solis and Wets local search methods. Initial positions, orientations, and torsions of the ligand molecules were set randomly. All of the rotatable torsions were released during docking. Each run of the docking experiment was set to terminate after a maximum of 2.5 × 10^6^ energy evaluations. The population size was set to 150. During the search, a translational step of 0.2 Å and quaternion and torsion steps of five were applied. The docking figures for publication were prepared in Discovery Studio 4.0 (Accelrys Software Inc., 2013) [42].

### 3.9. Cytotoxicity

#### 3.9.1. Cell Lines and Culture Conditions

Human cancer cell lines HepG2 (Liver), SK-MEL-1 (Skin), HT 018 (Colon), HeLa (Cervical), and MDA-MB 231 (Breast) were procured from the American Type Culture Collection (Rockville, MD, USA). MDA-MB-231 and HepG2 cell lines were maintained in DMEM; whereas SK-MEL-1, HeLa, and HT-018 were maintained in RPMI medium. The complete growth medium was supplemented with 10% (*v*/*v*) heat-inactivated FCS, 2 mM l-glutamine, and antimycotic-antibiotic solution. All of the cells were maintained in a standard culture condition of 37 °C temperature and 95% humidified atmosphere containing 5% CO_2_. Cells were screened periodically for mycoplasma contamination.

#### 3.9.2. MTT Assay

The novel complexes **1** and **2** were examined for their cytotoxicity against five different types of cancer cell lines viz., HepG2, SK-MEL-1, HT 018, HeLa, and MDA-MB 231 using a standard MTT reduction assay, according to Khan et al. (2012) [35]. Briefly, cells at a concentration of 1 × 10^6^ cells/200 mL/well were plated in 96-well plates and further grown in their respective medium containing 10% FCS. After 24 h, cells were incubated with 0–25 μM concentrations of test complexes or respective free ligands. Cells with 0.1% DMSO (vehicle control) and cisplatin (positive control) were also cultured under the same conditions. Cells only were used as negative control. Ensuing 48 h of incubation, old medium from treated cells was replaced with fresh medium. MTT reagent (5 mg/mL in PBS) was added to each well, and cells were further incubated for 2–3 h at 37 °C. After treatment, the supernatants were carefully removed, and 100 μL of DMSO was added to each well. Absorbance was measured at 620 nm in a multi-well plate reader, and drug sensitivity was expressed in terms of the concentration of drug required for a 50% reduction of cell viability (IC_50_).

#### 3.9.3. Measurement of Cancer Cell Adhesion

Assay for adhesion was performed with cytoselect 24-well cell adhesion as per protocol [43]. For quantitative analysis, the IC_50_ concentration of respective complexes (in triplicate) was tested on HepG2, SK-MEL-1, HT 018, HeLa, and MDA-MB 231 cancer cells. Cancer cell (0.1–1.0 × 10^6^ cells/mL) suspension in serum-free medium was added to the inside of each well of a pre-warmed adhesion plate. The plates were incubated for 30–90 min in a CO_2_ incubator and treated as per the protocol [43]. After air-drying the wells, stained adhered cells were extracted using an extraction solution. Then, the absorbance of the extracted sample (adhered cells) was read at 560 nm in a microtiter plate reader.

#### 3.9.4. Measurement of Cancer Cell Migration

The cell migration assay was performed with a cytoselect 24-well cell adhesion assay according to the protocol (Khan et al., 2013) [43] on HepG2, SK-MEL-1, HT 018, HeLa, and MDA-MB 231 cancer cell lines. For quantitative analysis, the IC_50_ concentration of the respective complexes (in triplicate) was tested on HepG2 (Liver), SK-MEL-1 (Skin), HT 018 (Colon), Hela (Cervical), and MDA-MB 231 (Breast) cancer cells. The test complexes were supplemented with medium containing 10% FBS in the lower well of the migration plate. To the inside of each insert, 100 μL of 0.5–1.0 × 10^6^ cells/mL of cell suspension was added. The plates were then incubated for 8 h at 37 °C in a humidified CO_2_ incubator. Finally, the absorbance of 100 μL of each sample was then read at 560 nm.

#### 3.9.5. Analysis of Annexin-V Binding by Flow Cytometry

Annexin-V staining was performed according to the protocol (Khan et al., 2017) [44]. For quantitative analysis, the IC_50_ concentration of respective complexes (in triplicate) was tested on HepG2 (Liver), SK-MEL-1 (Skin), HT 018 (Colon), Hela (Cervical), and MDA-MB 231 (Breast) cancer cells. Cancer cell (0.1–1.0 × 10^7^ cells/mL) suspension in serum-free medium was incubated with respective complexes in six-well plates in a CO_2_ incubator. After treating with complexes for 48 h, the cancer cells were harvested and incubated with annexin V-FITC and propidium iodide (PI). The fluorescence emission of Annexin-V stained cells was measured at 530–575 nm in a flow cytometer (MACSQuant, Bergisch Gladbach, Germany). Dots represent cells as follows: lower left quadrant, normal cells (FITC^−^/PI^−^); lower right quadrant, early apoptotic cells (FITC^+^/PI^−^); upper left quadrant, necrotic cells (FITC^−^/PI^+^); upper right quadrant, late apoptotic cells (FITC^+^/PI^+^).

### 3.10. In Vivo Toxicity

#### 3.10.1. Toxicity Study Design

Mice (males) were randomly divided into different groups (*n* = 6–10). Different doses of complexes **1** and **2** (0.5, 1, 2, 5, 8 and 10 g/kg) were administered intraperitoneally. The complexes were suspended in 0.2% aqueous Tween 80 or 0.25% carboxymethylcellulose. The animals were observed for 72 h for signs of toxicity and mortality, and LD_50_ was calculated according to a published method [45].

#### 3.10.2. Chronic Toxicity Study

A total of 40 male and 40 female Swiss albino mice were randomly allocated to the control and test groups. The complexes 1 and 2 in each case were mixed with drinking water for the feasibility of administration due to long treatment duration. The dose selected was 1/10^th^ of the LD_50_. The treatment was continued for a period of 12 weeks [46] (WHO Scientific Group, Geneva, 1967). The animals were then observed for all external general symptoms of toxicity, body weight changes, and mortality. Ten male and 10 female rats were used in each group, having one control and two treated groups. One group of treated female rats was mated with treated males, and pregnancy outcomes were studied. Urine was collected 1–2 days before the end of the treatment. The treated animals were fasted for 12 h and then anesthetized with ether. Blood samples were collected via heart puncture and centrifuged at 3000 rpm for 10 min. The plasma was then stored at −20 °C pending for analysis of the biochemical parameters. Vital organs were removed, weighed, and investigated for apparent signs of toxicity, and stored in 10% formalin for histological studies. The percentage of each organ relative to the body weight of the animal was calculated.

#### 3.10.3. Hematological Studies

Whole non-centrifuged blood was used for the determination of some hematological parameters. The blood was analyzed for WBC and RBC count, hemoglobin, platelets, neutrophils, and lymphocytes measurements using Contraves Digicell 3100H (Zurich, Switzerland) [47].

#### 3.10.4. Serum Analysis of Biochemical Parameters

A colorimetric method was used for the determination of the biochemical parameters (SGPT, SGOT, GGT, ALP, bilirubin, and lipid profile) in plasma. The enzyme activity was quantified spectrophotometrically using commercial enzymatic kits (Crescent diagnostics test kits, Jeddah, KSA).

#### 3.10.5. Statistical Analysis

The results were presented as a mean ± standard error of the mean (S.E.M). Statistical differences were analyzed using ANOVA with the Dunnett test as a post-hoc test. A value of *p* < 0.05 was considered statistically significant [48].

## 4. Conclusions

The new copper(II) (1) and zinc(II) (2) complexes have been designed and prepared with the benzimidazole-derived ligand as a biologically active moiety. The focus of our work was on its biological significance, in particular its anticancer property. Thus, complexes 1 and 2 were studied to examine the propensity of binding with HSA and DNA, which confirmed the avid binding. DNA cleavage experiments showed the evident nuclease activity of complex 1 with a double-stranded cleavage mechanism, and further revealed that ROS were responsible for the cleavage activity. Interestingly, the cytotoxicity of both the complexes and the ligand was examined on the five different cancer cells line, and the results showed that the activity of complex 1 was considerably good on breast cancer cells, with the IC_50_ values comparable to the standard drug cisplatin. Furthermore, we have studied the cell adhesion and cell migration properties of different cancer cell line in the presence of complex 1. Our findings exhibited that complex 1 showed significant anti-metastatic potential by inhibiting the adhesion and migration property of cancer cells. Moreover, the in vivo chronic toxicity of both complexes 1 and 2 revealed that they were safe and could be developed as a potential anticancer drug for human consumption. Overall, the results of this study ascertain that copper complex (1) can be a promising chemotherapeutic intervention for future application in cancer therapy, but warrants further investigations.

## Figures and Tables

**Table 1:** Quenching constants and binding parameters for HSA–metal complex interactions.

**Binding Parameters**					
**Complex**	**Temp**	***K*_SV_**	***k_q_***	***n***	***K_b_***
	**(K)**	**×10^4^ (M^−1^)**	**×10^12^ (M^−1^ s^−1^)**		**×10^4^ (M^−1^)**
**1**	298	2.02	3.54	1.09	1.58
	303	1.75	3.24	1.12	1.27
	308	1.51	2.64	1.20	0.86
**2**	298	2.74	4.79	0.99	2.78
	303	2.39	4.19	1.03	2.19
	308	1.97	3.45	1.11	1.48
**Thermodynamic Parameters**					
**Complex**	**Temp**	**∆*H***	**∆*S***	***T*∆*S* (kcal/mol)**	**∆*G***
	**(K)**	**(kcal/mol)**	**(cal/mol/K)**		**(kcal/mol)**
**1**	298	−11.12	−18.17	−5.41	−5.71
	303			−5.50	−5.62
	308			−5.60	−5.52
**2**	298	−11.48	−18.14	−5.40	−6.08
	303			−5.49	−5.99
	308			−5.59	−5.89
**FRET Parameters**					
**Complex**	***J***		***R_o_***	***R***	
	**×10^−15^ (M^−1^ cm^−1^)**		**(nm)**	**(nm)**	
**1**	3.26		2.04	2.35	
**2**	4.63		2.16	2.36	

**Table 2:** List of residues of HSA interaction with different complexes.

Interaction	Nature of Interaction	Bond Length (Å)	Binding Affinity, *K_d_* (M^−1^)	Δ*G* (kcal/mol)
Complex 1				
Unk:C—Asp451:O^δ2^	Hydrogen Bond	3.71	7.8 × 10^6^	−9.4
Lys199:C^β^—Unk	Hydrophobic (π-σ)	3.89		
Trp214—Unk	Hydrophobic (π-π)	4.79		
His242—Unk	Hydrophobic (π-π)	5.25		
Unk—Trp214	Hydrophobic (π-π)	4.91		
Unk—Arg218	Hydrophobic (π-alkyl)	5.23		
Unk—Lys195	Hydrophobic (π-alkyl)	4.92		
**Complex 2**				
Unk:C—His242:N^e2^	Hydrogen Bond	3.25	4.2 × 10^7^	−10.4
Unk:C—Ala291:O	Hydrogen Bond	3.62		
Lys199:C^β^—Unk	Hydrophobic (π-σ)	3.68		
Ala291:C^β^—Unk	Hydrophobic (π-σ)	3.59		
His242—Unk	Hydrophobic (π-π)	4.86		
His242—Unk	Hydrophobic (π-π)	4.69		
Unk—Trp214	Hydrophobic (π-π)	5.05		
Unk—Lys195	Hydrophobic (π-alkyl)	5.02		
Unk—Leu238	Hydrophobic (π-alkyl)	5.15		

**Table 3:** Molecular interactions between DNA and complexes **1** and **2**.

Interaction	Bond Distance (Å)	Nature of Interaction	*E*_total_ Value
Complex 1			
Unk:H—B:d21:O2	2.57	Hydrogen Bond	−238.14
Unk:H—B:dG22:O4′	2.25	Hydrogen Bond	
Unk:H—A:dA5:N3	2.34	Hydrogen Bond	
Unk:H—A:dA5:N3	2.98	Hydrogen Bond	
Unk:H—B:d21:O2	2.79	Hydrogen Bond	
B:d23:OP1—Unk	4.63	Electrostatic (π-Anion)	
**Complex 2**			
Zn—B:dA18:OP1	5.41	Electrostatic	−248.34
Unk:H—A:dG10:O3′	2.01	Hydrogen Bond	
Unk:H—A:d11:O4′	2.89	Hydrogen Bond	
Unk:H—A:dG10:OP1	2.93	Hydrogen Bond	
Unk:H—B:dA18:O3′	2.62	Hydrogen Bond	
Unk:H—B:dT19:OP1	2.81	Hydrogen Bond	
B:dA18:OP1—Unk	2.88	Electrostatic (π-Anion)	
B:dA18:OP2—Unk	4.88	Electrostatic (π-Anion)	
B:dA17:O3′—Unk	2.87	π-Lone Pair	
B:dA17:O3′—Unk	2.55	π-Lone Pair	

**Table 4:** IC_50_ values of complexes **1**, **2**, the ligand, and cisplatin against five human cancer cell lines.

Complex	HepG2	SK-MEL-1	HT018	HeLa	MDA-MB 231
	(Liver)	(Skin)	(Colon)	(Cervical)	(Breast)
	(μM)	(μM)	(μM)	(μM)	(μM)
1	14 ± 2.2	17.8 ± 2.7	15 ± 2.1	13 ± 2.2	3.5 ± 2.4
2	19 ± 2.6	18 ± 2.3	25 ± 4.0	24.5 ± 2.2	26.7 ± 4.1
Ligand	NA	NA	NA	NA	NA
Vehicle control (0.1% DMSO)	NA	NA	NA	NA	NA
Cisplatin (Positive control)	6 ± 0.4	5.6 ± 0.8	5.7 ± 0.2	6 ± 0.6	3.1 ± 0.2

NA stands for Not Active.

## Supplementary Materials

The following are available online at www.mdpi.com/1422-0067/19/5/1492/s1.
