# Supplementary material for: Design, Synthesis, and Biological Evaluation of Benzimidazole-Derived Biocompatible Copper(II) and Zinc(II) Complexes as Anticancer Chemotherapeutics
Source: Int J Mol Sci. 2018 May 16;19(5):1492. doi: 10.3390/ijms19051492 (PMC5983663; doi:10.3390/ijms19051492)
Supplement: Supplementary file 1 [file ijms-19-01492-s001.pdf]

Supplementary Information

Design, Synthesis, and Biological Evaluation of Benzimidazole-derived Biocompatible Copper(II) and Zinc(II) Complexes as Anticancer Chemotherapeutics

Mohamed F. AlAjmi[1], Afzal Hussain[1*], Md T. Rehman[1], Azmat A. Khan[2], Pervez A.Sheikh[1], and Rais A. Khan [3*]

[1]Department of Pharmacognosy, College of Pharmacy, King Saud University, P.O. Box 2457, Riyadh 11451, KSA; malajmii@ksu.edu.sa (M.F.A.); afzal.hussain.amu@gmail.com (A.H.); m.tabish.rehman@gmail.com (M.T.R.); aperwez@ksu.edu.sa (P.A.S.)
[2]Department of Pharmaceutical Chemistry, College of Pharmacy, King Saud University, P.O. Box 2457, Riyadh 11451, KSA; azmatbiotech@gmail.com (A.A.K.)
[3]Department of Chemistry, College of Science, King Saud University, P.O. Box 2455, Riyadh 11451, KSA; raischem@gmail.com (R.A.K.)
[*]Corresponding author: raischem@gmail.com , Tel: +966 536745404; afzal.hussain.amu@gmail.com , Tel: +966504767847

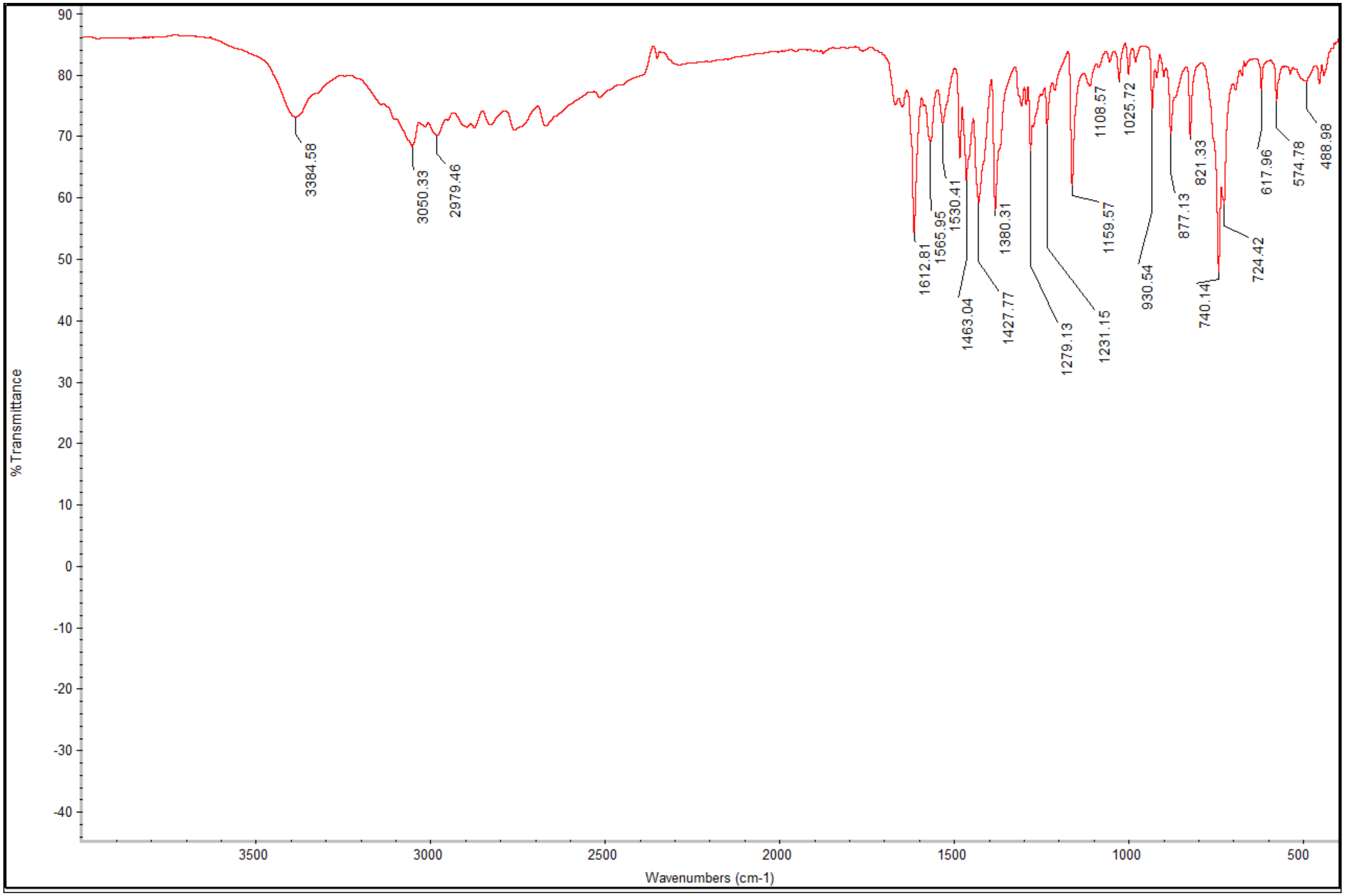

Figure S1. Fourier transform infrared (FT-IR) spectrum of the ligand "BnI".

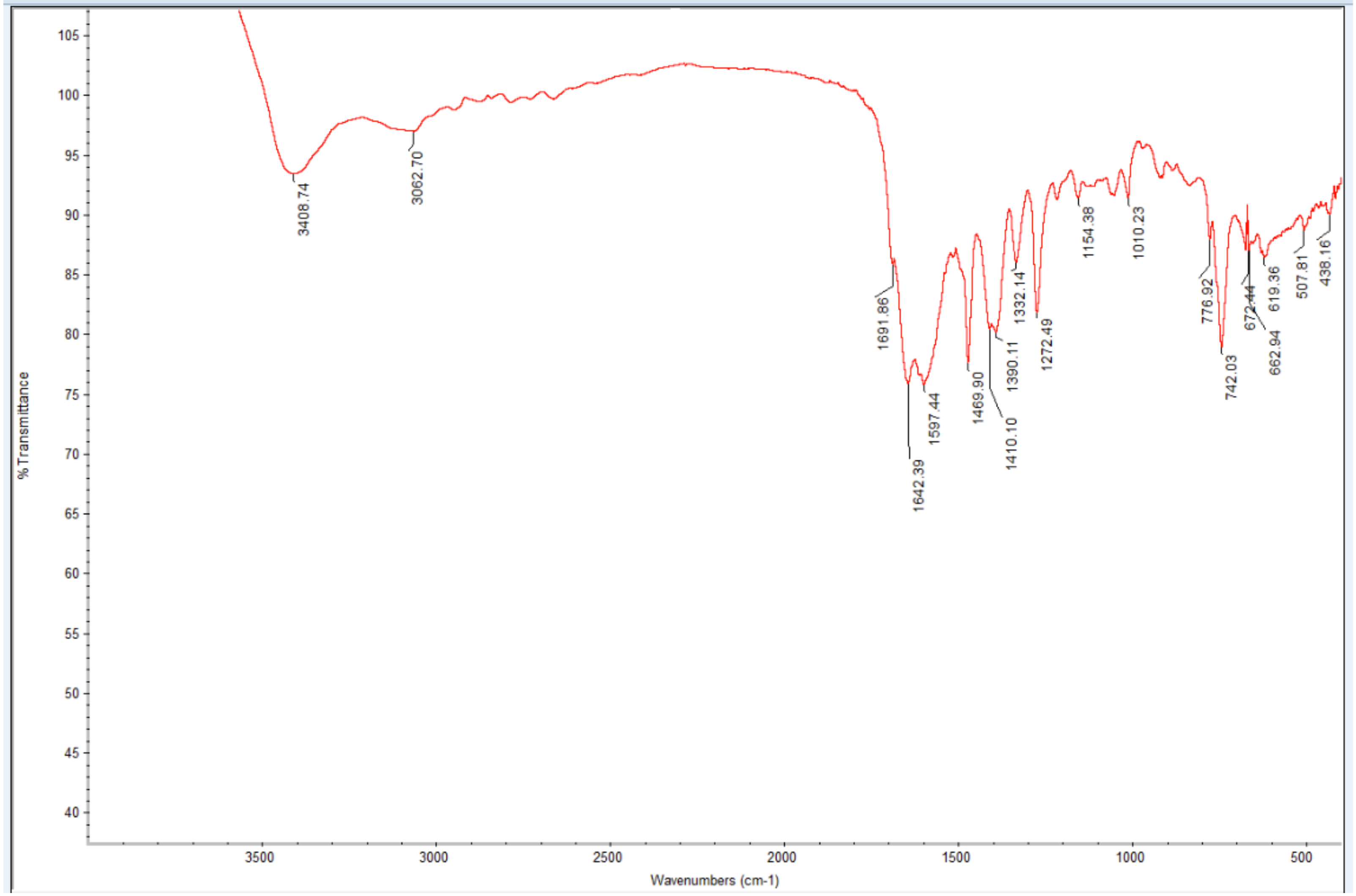

Figure S2. FT-IR spectrum of the copper complex (**1**).

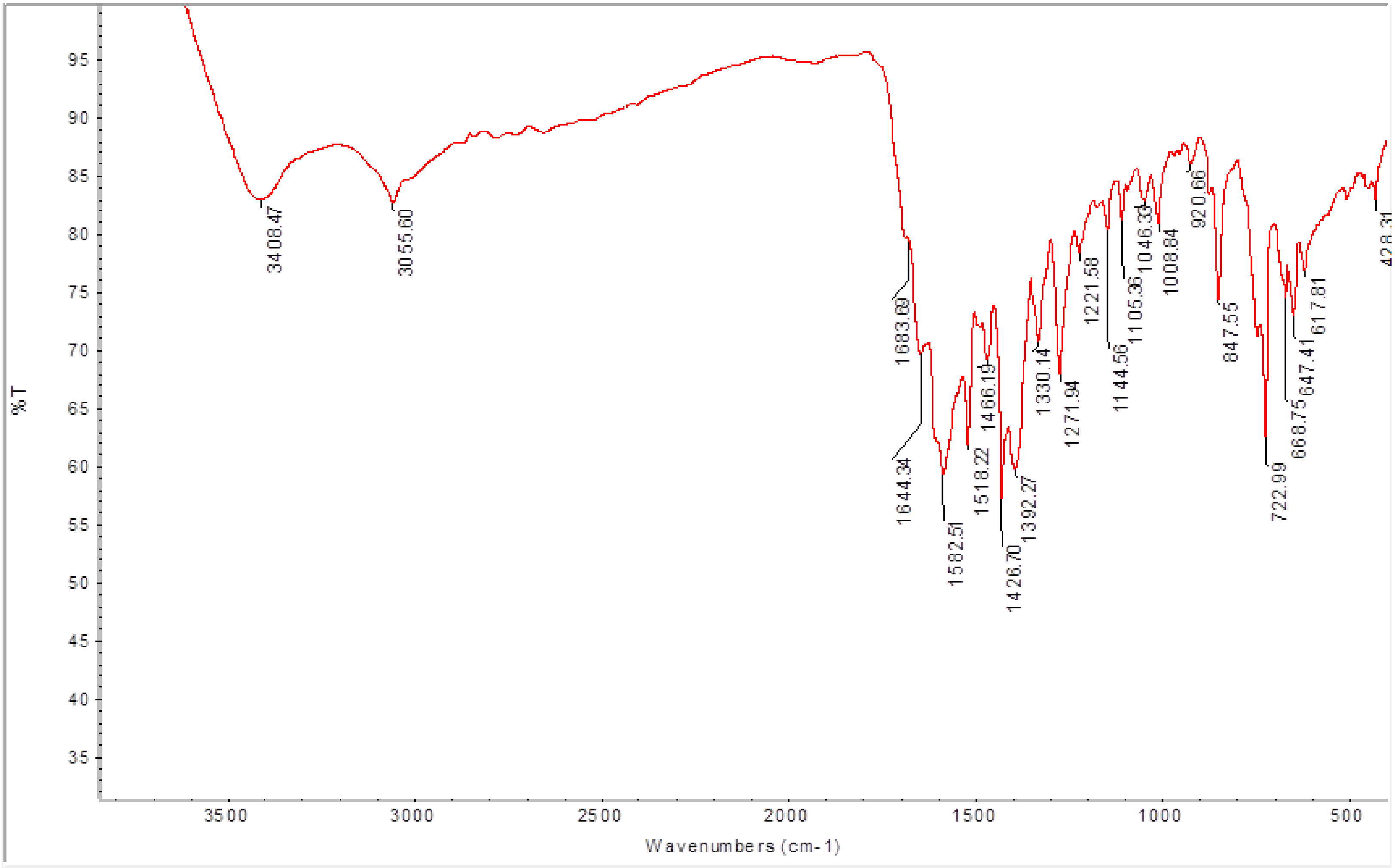

Figure S3. FT-IR spectrum of the zinc(II) complex (**2**).

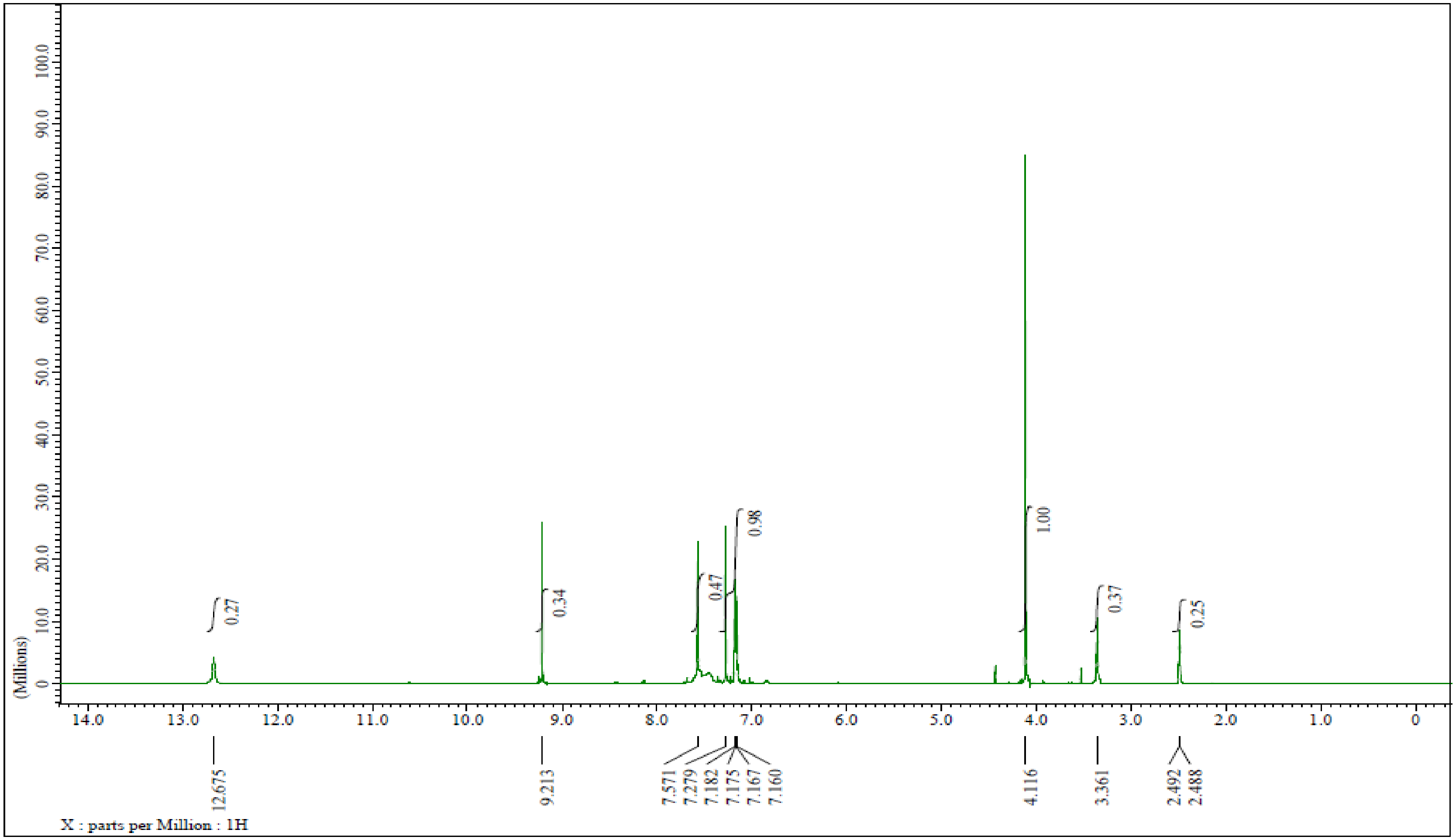

Figure S4. ^{1}H NMR spectrum of the ligand.

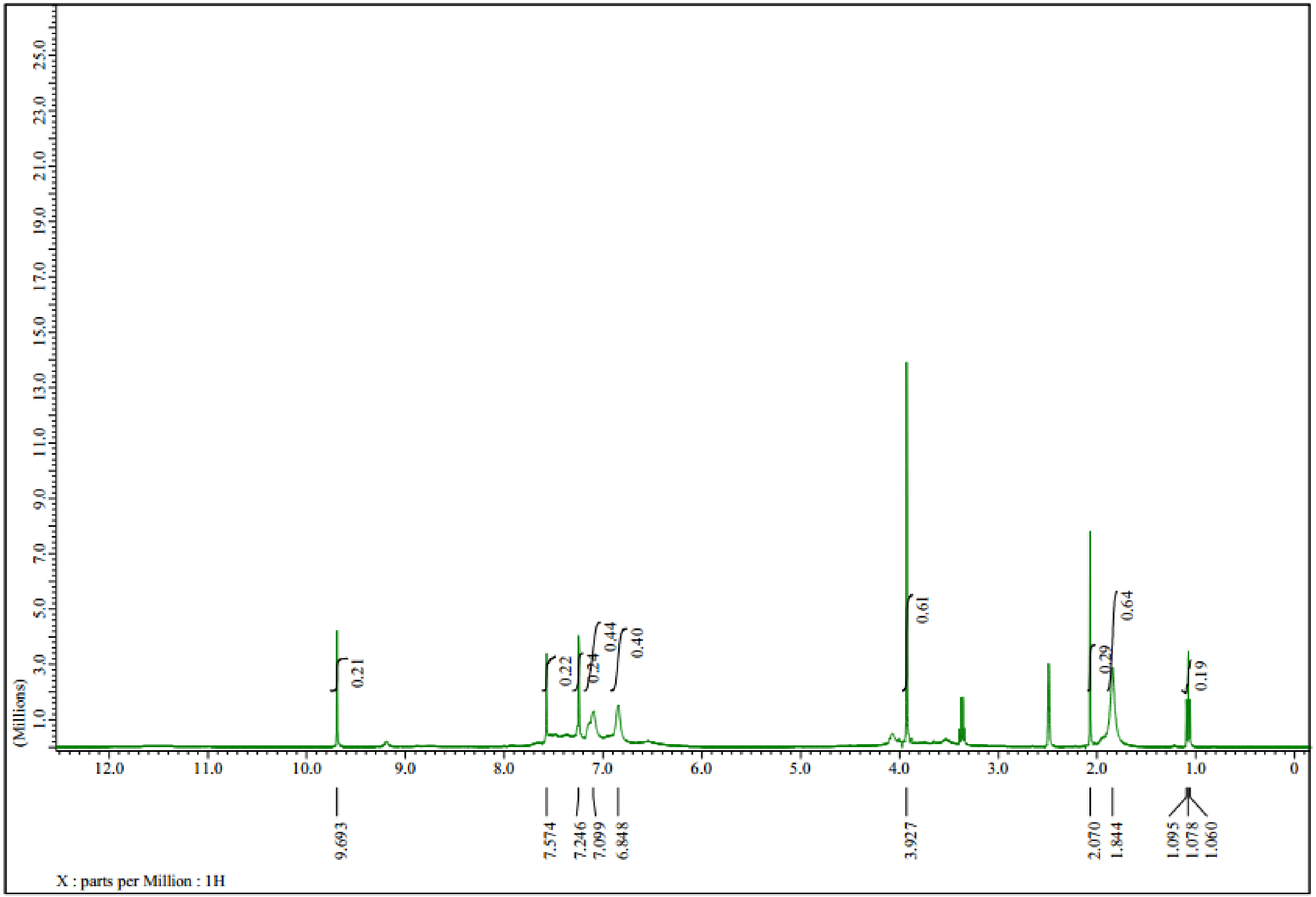

Figure S5. ^{1}H NMR spectrum of complex **2**.

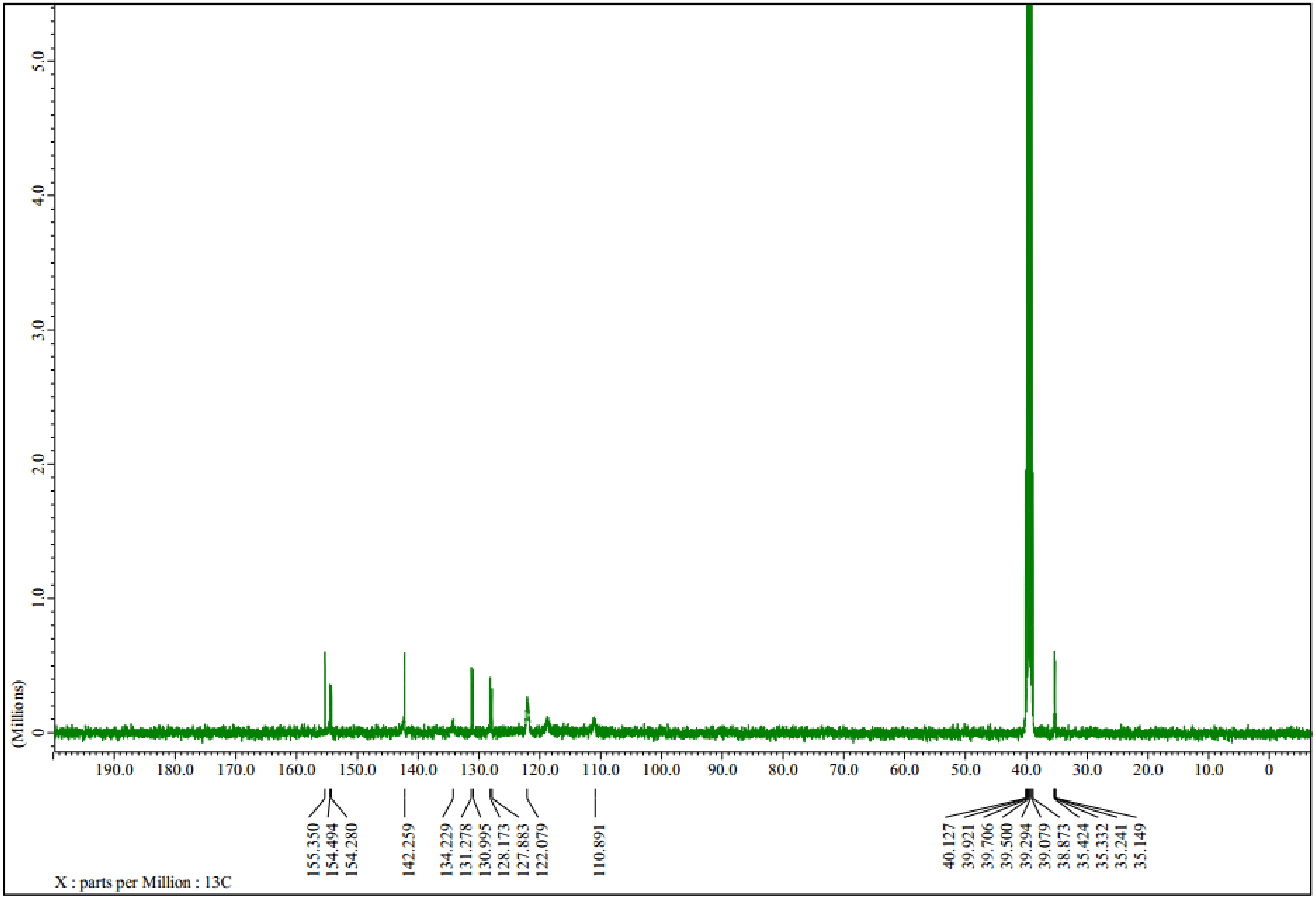

Figure S6. ^{13}C NMR spectrum of the ligand.

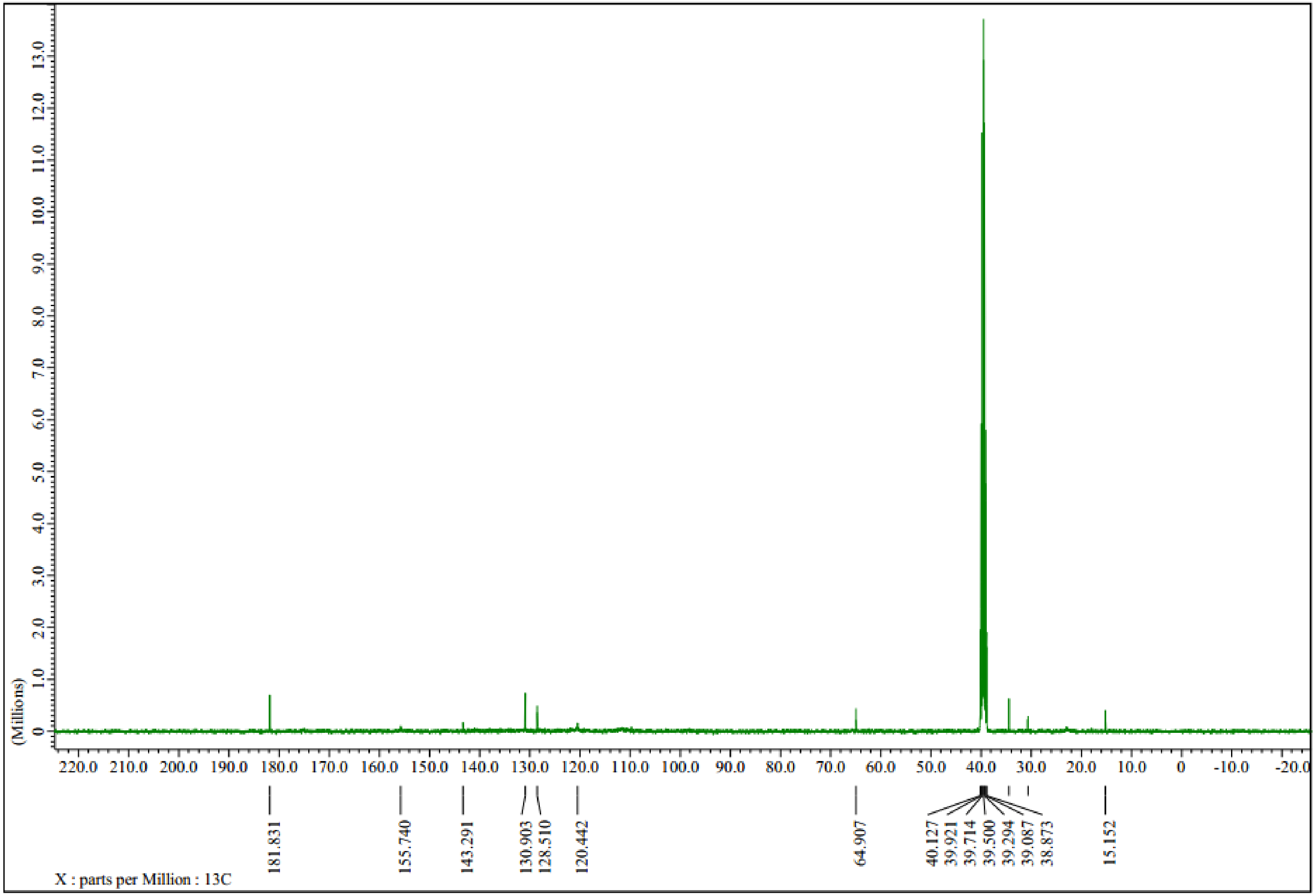

Figure S7. ^{13}C NMR spectrum of complex **2**.
